# The frequency of tetracycline resistance genes co-detected with respiratory pathogens: a database mining study uncovering descriptive trends throughout the United States

**DOI:** 10.1186/1471-2334-14-460

**Published:** 2014-08-25

**Authors:** Matthew D Huff, David Weisman, John Adams, Song Li, Jessica Green, Leslie L Malone, Scott Clemmons

**Affiliations:** Diatherix Laboratories Inc., 601 Genome Way, Suite 2100, Huntsville, Al 35806 USA; Department of Biology, University of Massachusetts Boston, 100 Morrissey Blvd., Boston, MA 02125-3393 USA; Knoxville Infectious Disease Consultants, P.C., 2210 Sutherland Ave., Suite 110, Knoxville, TN 37919 USA; Hudson Alpha Institute of Biotechnology, 601 Genome Way, Huntsville, AL 35806 USA

## Abstract

**Background:**

The Center for Disease Control and Prevention (CDC) indicates that one of the largest problems threatening healthcare includes antibiotic resistance. Tetracycline, an effective antibiotic that has been in use for many years, is becoming less successful in treating certain pathogens. To better understand the temporal patterns in the growth of antibiotic resistance, patient diagnostic test records can be analyzed.

**Methods:**

Data mining methods including frequent item set mining and association rules via the Apriori algorithm were used to analyze results from 80,241 Target Enriched Multiplex-PCR (TEM-PCR) reference laboratory tests. From the data mining results, five common respiratory pathogens and their co-detection rates with tetracycline resistance genes (TRG) were further analyzed and organized according to year, patient age, and geography.

**Results:**

From 2010, all five pathogens were associated with at least a 24% rise in co-detection rate for TRGs. Patients from 0–2 years old exhibited the lowest rate of TRG co-detection, while patients between 13–50 years old displayed the highest frequency of TRG co-detection. The Northeastern region of the United States recorded the highest rate of patients co-detected with a TRG and a respiratory pathogen. Along the East–west gradient, the relative frequency of co-detection between TRGs and respiratory pathogens decreased dramatically.

**Conclusions:**

Significant trends were uncovered regarding the co-detection frequencies of TRGs and respiratory pathogens over time. It is valuable for the field of public health to monitor trends regarding the spread of resistant infectious disease, especially since tetracycline continues to be utilized a treatment for various microbial infections. Analyzing large datasets containing TEM-PCR results for co-detections provides valuable insights into trends of antibiotic resistance gene expression so that the effectiveness of first-line treatments can be continuously monitored.

**Electronic supplementary material:**

The online version of this article (doi:10.1186/1471-2334-14-460) contains supplementary material, which is available to authorized users.

## Background

In 1948, tetracycline was one of the first antibiotics discovered that disrupted ribosomal subunit assembly in bacterial pathogens. Tetracycline was advantageous over existing agents because the medication was inexpensive and had broad-spectrum response, but with a lower level of allergenic properties [1]. As a result, physicians began to prescribe the antibiotic to patients for many bacterial infections [2]. Over time, tetracycline appeared in agriculture, as farmers learned to fortify animal feeds with the antibiotic as it promoted growth and decreased the spread of bacterial pathogens [3–6]. Tetracycline was also sprayed on crops to halt the transmission of plant diseases [7]. By 1990, the rampant utilization of tetracycline had resulted in an increase in the number of tetracycline resistant infections.

The United States Centers for Disease Control and Prevention (CDC) has identified antibiotic resistance as one of the largest problems threatening global health. Antibiotic resistance directly costs healthcare over $20 billion annually; after including lost productivity, the total costs exceed $35 billion [8]. These enormous costs of tetracycline resistance may be largely unnecessary, as over 50% of the antibiotics prescribed are unnecessary [8]. The CDC, The United States Food and Drug Administration (FDA), and The United States Department of Agriculture are trying to modify policies regarding the prescription of medical antibiotics and their usage in agriculture to attempt to halt the growth of antibiotic resistance [9]. However, agriculturalists are reluctant to follow these policy changes because of a lack of factual evidence linking excess usage to rising levels of resistance. Even if this aspect of tetracycline resistance is widely disputed, the CDC strongly argues that tracking trends in existing antibiotic infections is a key weapon in preventing new antibiotic resistant infections.

Target Enriched Multiplex-Polymerase Chain Reaction (TEM-PCR™) (Diatherix Laboratories Inc. Huntsville, AL) has the ability to simultaneously detect Methicillin Susceptible *Staphylococcus aureus* (*S.aureus*), Methicillin Resistant *Staphylococcus aureus* (MRSA), *Streptococcus pneumoniae* (*S. pneumoniae*), *Haemophilus influenzae* (*H. influenzae*), *Moraxella catarrhalis* (*M. catarrhalis*), and tetracycline resistance genes (TRG) tet (K) and tet (M) [10–14]. TEM-PCR is a nucleic acid multiplex technology that includes two main amplification steps: target enrichment and subsequent exponential amplification [10–14]. For the target enrichment step, two pairs of nested gene-specific primers (*Fo,* forward out; *Fi,* forward in; *Ri* reverse in, *Ro* reverse out) are designed for each target. Low concentrations of the nested primers are used to amplify the target DNA, which will be exponentially amplified in the next step. The *Fi* and *Ri* primers contain a unique tag sequence that can be recognized by a universal set of forward and biotin-labeled reverse SuperPrimers. In the exponential amplification step, the SuperPrimers are present at a concentration that allows for asymmetric PCR amplification of the biotin-labeled single-stranded DNA targeted by the reverse SuperPrimer. Target-specific detection probes are then coupled to carboxyl groups on a bead-based substrate, and probe fluorescence is read using a bead-based detection platform.

Previous literature has documented the rise in tetracycline resistant pathogens and warned of the possible medical consequences regarding antimicrobial immunity [1, 2, 15–17]. This descriptive study analyzed a dataset of 80,421 patient TEM-PCR test results, and explored the co-detections between tetracycline resistance genes and the respiratory pathogens. By elucidating these co-detection patterns, the study contributes to the knowledge surrounding tetracycline resistance and aids in accomplishing the ultimate goals of optimizing patient care and reducing unnecessary healthcare costs.

## Methods

### TEM-PCR

Nucleic acid extraction for TEM-PCR was performed using a KingFisher™ system (Thermo Scientific). The amplification steps were completed using a GeneAmp® PCR System 9700 thermocycler (Applied Biosystems). Probe fluorescence was read using a Luminex detection platform. TEM-PCR is a proprietary testing assay with detailed procedures and SOPs under the ownership of Diatherix Laboratories Inc.

### Data mining methods

The two techniques used to elucidate relationships in these data are *frequent item sets* and *association rules* via the Apriori algorithm [18–22]. Briefly, the Apriori algorithm is a widely used algorithm that is commonly applied to transactional datasets. Frequent item sets are groups of DNA markers that occur more frequently than a minimum *support* level in the database. For example, in this dataset, TRG and *S. pneumoniae* were co-detected in 19.8% of the tests that identified at least one marker, which is above the minimum support count of 50 positive tests. The Apriori algorithm assembles frequent item sets into association rules that indicate conditional frequency. For example, the rule {A, B} = > {C} indicates that when A and B are found, C is also frequently found. The *confidence* of an association rule indicates its conditional probability in the dataset, that is P(C | A,B). A rule’s interestingness measure *lift* indicates the degree of independence between the antecedent and the consequent, with values greater than 1 indicating an interesting rule.

Full details of support count, support, confidence, and lift can be found in publications [23, 24]. Data mining methods described here were recently used in many biomedical publications [19, 23–26]. One publication that clearly explains the methods applied in this study includes a paper by Naulaerts et al. [27]. The confidence, support, lift, and support count values from the Apriori algorithm promoted further co-detections analysis of the 5 selected respiratory pathogens and TRGs.

### Retrospective co-detection analysis of reference laboratory data

Using reference laboratory TEM-PCR data, 80,241 patient tests results were analyzed for the detection of *S. aureus*, MRSA, *S. pneumoniae, H. influenzae, M. catarrhalis,* and TRGs. 49,840 (62.1%) tests were detected for one pathogen or TRG. The co-detection rates between each pathogen and TRG were further analyzed by year, patient age by year, and geography. In all categorical subsets, results were analyzed for all respiratory samples from November 9, 2009-August 7, 2013. The analysis of *M. catarrhalis* began on September 30, 2011 after genetic targets for this pathogen were added to the multiplexing assay. Data reflecting multiple samples assayed from a given patient on a single date were removed from the analysis. The sample size for each year was within the range of 6,396 (2009) < X < 26,841 (2012). Patient age was organized into 4 groups (0 (exclusive)-2 (inclusive)], (2–13], (13–50], and (50–100]. 91.1% of patients aged (0–2], 94.7% of patients aged (2–13], 92.4% patients aged (13–50], and 76.6% of patients aged (50–100] had specimen collected by nasal, nasalpharangeal, or throat swab. 8.7% of patients age (50–100] had sputum samples collected. Other specimen sources which represented a very small percentage of patients included aspirate, mucus, or bronchial or nasal washes. Results were included from each state where samples were collected within the sample size range of 60 (Maryland) < X < 16,985 (Tennessee). Patient sample size for each categorical subset including year, age group, and geographical state can be seen in Additional file 1: Tables S1-S7. Data analyzed comes from the frequency of patients that tested positive for the simultaneous presence of one pathogen and a TRG. Further molecular testing or bacterial plating by culture was not done to verify if these pathogens were the source of detected TRGs and further traditional testing was out of the scope of this study. Inclusion criteria were defined as submission for respiratory pathogen testing, without regard to age, demographic categories, or other medical conditions.

All of the database information was archived and organized using Microsoft SQL Server Management Studio 2012. Graphical analysis and statistics including scatter plots with loess smoothing, confidence intervals, variance, and heat maps were performed in the R language, with ggplot2 [28, 29]. Confidence intervals were at 95% confidence and constructed via the Jefferys prior [30]. Tables were generated in Microsoft Excel 2012. All reference laboratory tests were conducted at Diatherix Laboratories Inc. in Huntsville, AL.

## Results

The Apriori algorithm created a list of the 25 most frequent item sets found throughout the respiratory results database (Table 1). The largest measurements of support included TRGs (40.5%), *S. pneumoniae* (36.3%)*, H. influenzae* (28.7%), and *M. catarrhalis* (25.1%). *S. pneumoniae* and *H. influenzae* were co-detected with TRGs 19.8% and 15.1% of all positive tests results. MRSA and *S. aureus* were detected 12.4% and 10.0% of all positive results, respectively. 6.5% of all positive tests included co-detection for MRSA and TRGs and 4.1% included simultaneous detection between *S. aureus* and TRGs.

**Table 1:** **Frequent item sets**

Item sets	Support count	Support
**{Tetracycline}**	**20,207**	**40.54%**
**{** ***S. pneumoniae*** **}**	**18,113**	**36.34%**
**{** ***H. influenzae*** **}**	**14,281**	**28.65%**
**{** ***M. catarrhalis*** **}**	**12,491**	**25.06%**
**{** ***S. pneumoniae*** **, Tetracycline}**	**9,850**	**19.76%**
**{** ***H. influenzae*** **, Tetracycline}**	**7,543**	**15.13%**
**{** ***H. influenzae, S. pneumoniae*** **}**	**6,231**	**12.50%**
**{MRSA}**	**6,175**	**12.39%**
**{** ***S. pneumoniae, M. catarrhalis*** **}**	**5,864**	**11.77%**
**{Tetracycline,** ***M. catarrhalis*** **}**	**5,864**	**11.04%**
{CoxEchovirus1}	5,027	10.09%
**{** ***S. aureus*** **}**	**4,980**	**9.99%**
{Rhinovirus}	4,151	8.33%
**{** ***H. influenzae, M. catarrhalis*** **}**	**4,089**	**8.20%**
**{** ***H. influenzae, S. pneumoniae*** **, Tetracycline}**	**3,603**	**7.23%**
**{MRSA, Tetracycline}**	**3,066**	**6.15%**
{Parainfluenza}	3,027	6.07%
**{** ***S. pneumoniae*** **, Tetracycline,** ***M. catarrhalis*** **}**	**2,987**	**5.99%**
**{** ***H. influenzae*** **,** ***S. pneumoniae*** **,** ***M. catarrhalis*** **}**	**2,486**	**4.99%**
{*K.pneumoniae*}	2,160	4.33%
**{** ***S. aureus*** **, Tetracycline}**	**2,025**	**4.06%**
**{** ***H. influenzae*** **, Tetracycline,** ***M. catarrhalis*** **}**	**1,966**	**3.94%**
{Human Metapneumovirus}	1,955	3.92%
{*A.baumannii*}	1,801	3.61%

The first 25 frequent items with the highest support and support count are listed. The boldface rows indicate items that contain relationships between 1 or more of the pathogens or TRGs in this study.

From the frequent item set results, the Apriori algorithm identified the 25 association rules with the highest levels of support (Table 2). The top of the table includes the rules {S. pneumoniae} = > {Tetracycline}, {H. influenzae} = > {Tetracycline}, and {*H. influenzae, M. catarrhalis*} = > {*S. pneumoniae*} with 19.8%, 15.1%, and 7.2% support, respectively. Each of the highlighted association rules indicates a pathogen of interest or TRG that all have confidence measurements over 50.9% and lift values over 1.3. This confidence threshold establishes that, given the antecedent of a rule, the consequent is found in at least 50.9% of the cases. Since each rule had a lift measurement over 1, they were all considered to be rules of interest. From these results, and from literature associating TRGs with specific gram-positive bacteria, we sought to elucidate temporal co-detection patterns to better understand how patient age and geography relate to TRG co-detection.

**Table 2:** **Association rules**

Association rules	Support count	Support	Confidence	Lift
**{** ***S. pneumoniae*** **}= > {Tetracycline}**	**9,850**	**19.76%**	**54.38%**	**1.34**
**{** ***H. influenzae*** **}= > {Tetracycline}**	**7,543**	**15.13%**	**52.82%**	**1.30**
**{** ***H. influenzae*** **,** ***S. pneumoniae*** **}= > {Tetracycline}**	**3,603**	**7.23%**	**57.82%**	**1.43**
**{** ***S. pneumoniae*** **,** ***M. catarrhalis*** **}= > {Tetracycline}**	**2,987**	**5.99%**	**50.94%**	**1.26**
**{Tetracycline,** ***M. catarrhalis*** **}= > {** ***S. pneumoniae*** **}**	**2,987**	**5.99%**	**54.27%**	**1.49**
**{** ***H. influenzae*** **,** ***M. catarrhalis*** **}= > {** ***S. pneumoniae*** **}**	**2,486**	**4.99%**	**60.80%**	**1.67**
{*K. pneumoniae*}= > {Tetracycline}	1,518	3.05%	70.28%	1.73
**{** ***H. influenzae*** **,** ***S. pneumoniae*** **,** ***M. catarrahalis*** **}= > {Tetracycline}**	**1,311**	**2.63%**	**52.74%**	**1.30**
**{** ***H. influenzae*** **, Tetracycline,** ***M. catarrhalis*** **}= > {** ***S. pneumoniae*** **}**	**1,311**	**2.63%**	**66.68%**	**1.83**
{*A.baumannii*}= > {Tetracycline}	957	1.92%	53.14%	1.31
{CoxEchovirus1, *S.pneumoniae*}= > {Tetracycline}	888	1.78%	55.64%	1.37
{CoxEchovirus1, *S.pneumoniae*}= > {*S.pneumoniae*}	888	1.78%	55.88%	1.54
{*P.aeruginosa*}= > {Tetracycline}	812	1.63%	55.43%	1.36
{PVLgene}= > {MRSA}	805	1.62%	93.71%	7.56
**{MRSA,** ***S. pneumoniae*** **}= > {Tetracycline}**	**761**	**1.53%**	**61.56%**	**1.52**
{*S.pyogenes*}= > {Tetracycline}	689	1.38%	71.84%	1.77
**{** ***S. aureus*** **,** ***S. pneumoniae*** **}= > {Tetracycline}**	**651**	**1.31%**	**62.59%**	**1.54**
{CoxEchovirus1, *H.influenzae*}= > {Tetracycline}	646	1.30%	52.48%	1.29
{CoxEchovirus1, *H.influenzae*}= > {*S.pneumoniae*}	634	1.27%	51.50%	1.42
{CoxEchovirus1, *M.catarrahalis*}= > {*S.pneumoniae*}	627	1.26%	50.64%	1.39
{Rhinovirus, *S.pneumoniae*}= > {Tetracycline}	594	1.19%	51.65%	1.27
{Rhinovirus, Tetracycline}= > {*S.penumoniae*}	594	1.19%	53.66%	1.48
**{MRSA,** ***M. catarrhalis*** **}= > {Tetracycline}**	**574**	**1.15%**	**55.84%**	**1.37**
**{MRSA,** ***H. influenzae*** **}= > {Tetracycline}**	**556**	**1.12%**	**60.30%**	**1.49**
{PVLgene}= > {Tetracycline}	516	1.04%	60.00%	1.48

The first 25 association rules with the highest support and support count are listed. Other calculations include confidence and lift which have measurements over 52.8% and 1.3, respectively for all boldfaced rows.

**Figure 1:**
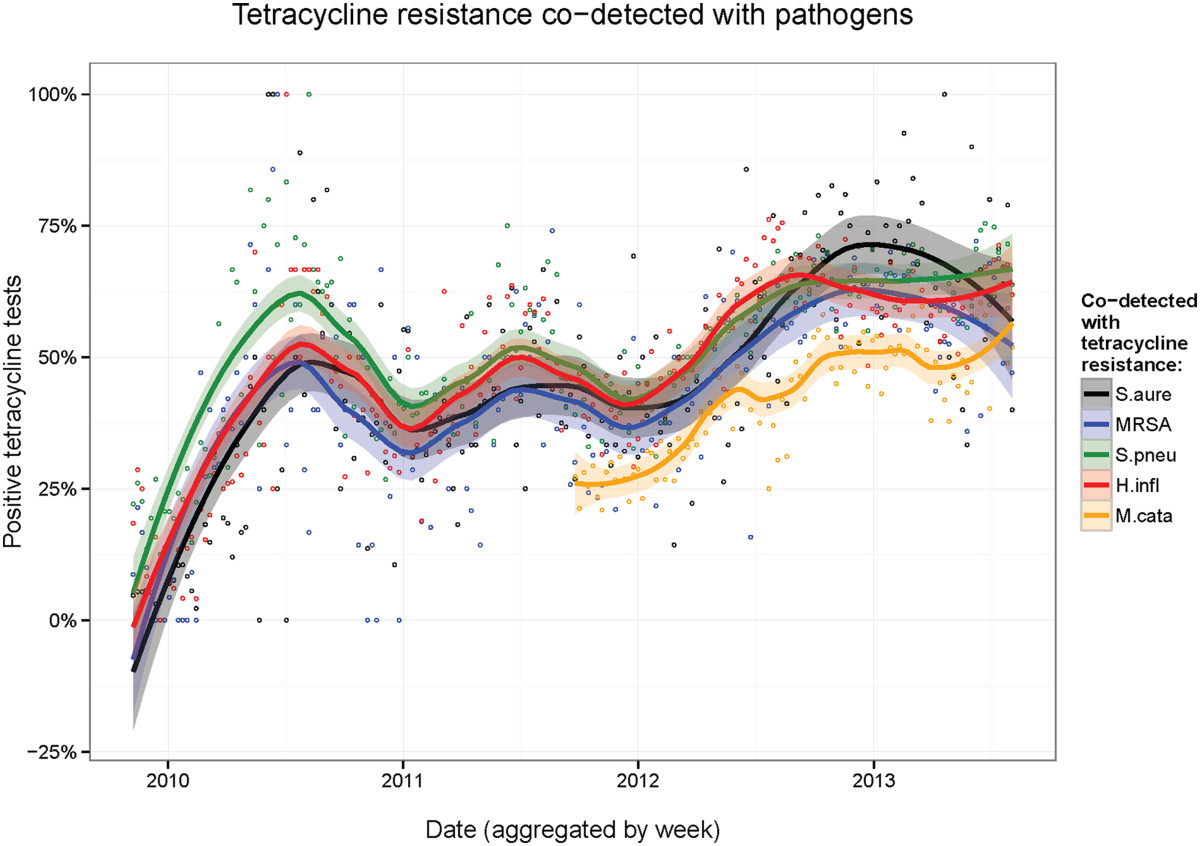
**The frequency of TRGs Detected with Respiratory Pathogens Increased from 2009–2013.** The scatterplot shows the rise in co-detection between TRGs and respiratory pathogens in recent years. The data is aggregated by week. Loess smoothing curves pass through the data points and are shaded by a 95% confidence interval.

Figure 1 displays a scatterplot including the frequencies of respiratory pathogens co-detected with TRGs by year. It is clear that the frequency of co-detection between each pathogen and TRG increased with time. From January 2010- August 2013, the percentage of gram-positive bacteria *S. aureus*, MRSA*,* and *S. pneumoniae* co-detected with TRGs rose 43%, 26%, and 27%, respectively. From January 2010- August 2013, the frequency of gram-negative bacteria *H. influenzae* and *M. catarrhalis* co-detected with TRGs rose 32% and 24%, respectively. Over each year, the rate of TRGs co-detected with these five potential pathogens rose a minimum of 3.9%.

**Figure 2:**
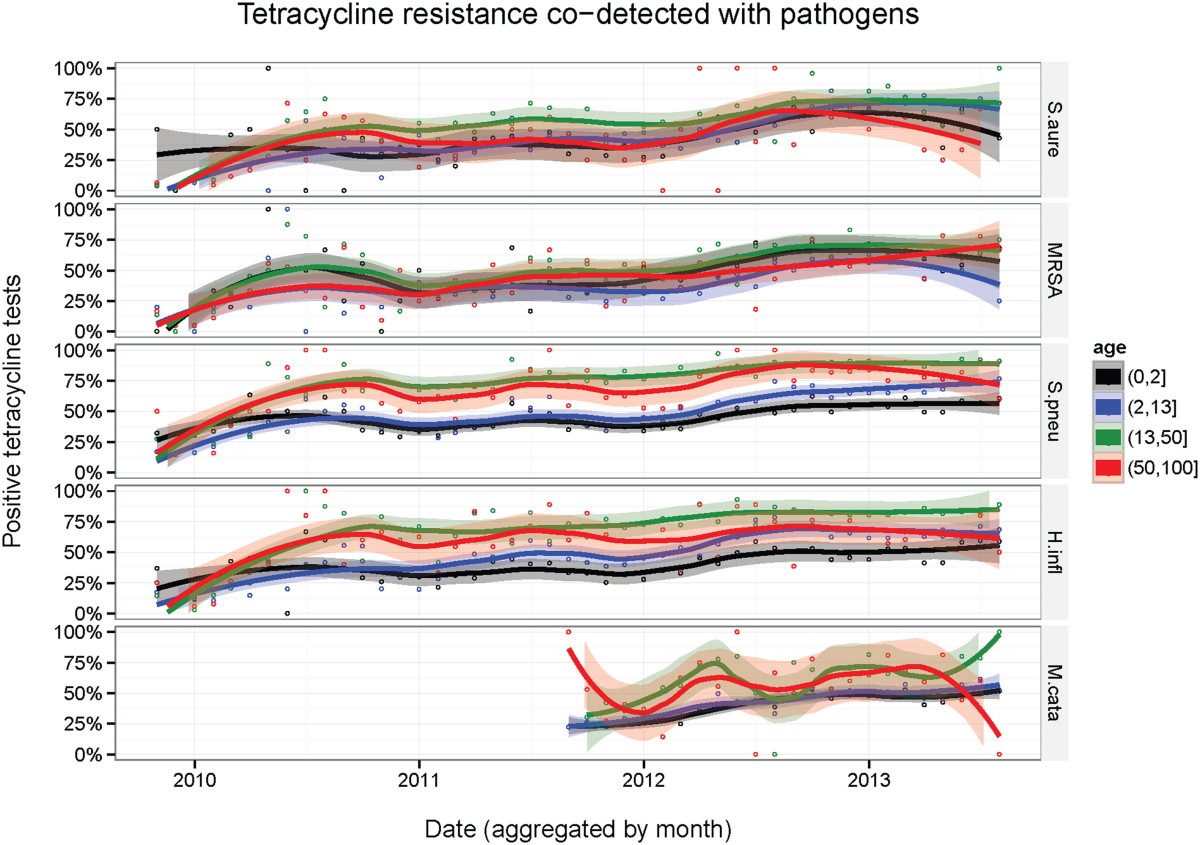
**The Frequency of TRGs Detected with Respiratory Pathogens Increased in Each Age Group over Time.** Each graph displays the co-detection frequency for each age group and pathogen. The data is aggregated by month. A loess smoothing line passes through data points and are shaded by 95% confidence intervals.

Figure 2 displays the time-series co-detection frequency of TRGs with each pathogen segmented by 4 age groups. The patient sample size and total number of positive tests for each pathogen are included in Additional file 1: Tables S1-S7. In each age group, the rates of co-detection between TRGs and each pathogen increasd with time. Older patients showed a larger co-detection rate than younger patients. For *H. influenzae*, *M. catarrhalis*, and *S. pneumoniae*, patients (0–2] had the lowest rate of co-detection, followed by patients (2–13] years of age, then patients (50–100] years of age, concluded by patients (13–50] years of age, which had the highest rate of co-detection. In 2013, there was an average 33%, 22%, and 26% difference in co-detection rate from children aged (0–2] to patients age (13–50] for *H. influenzae*, *M. catarrhalis*, and *S. pneumoniae*, respectively. In 2013, an alarming 82% patients of age (13–50] had TRGs co-detected with *S. pneumoniae*. For *S. aureus* and MRSA, the age group (13–50] had the highest rate of co-detection between TRGs and the designated pathogen, but all other age groups had similar and co-detection frequencies over time.

**Figure 3:**
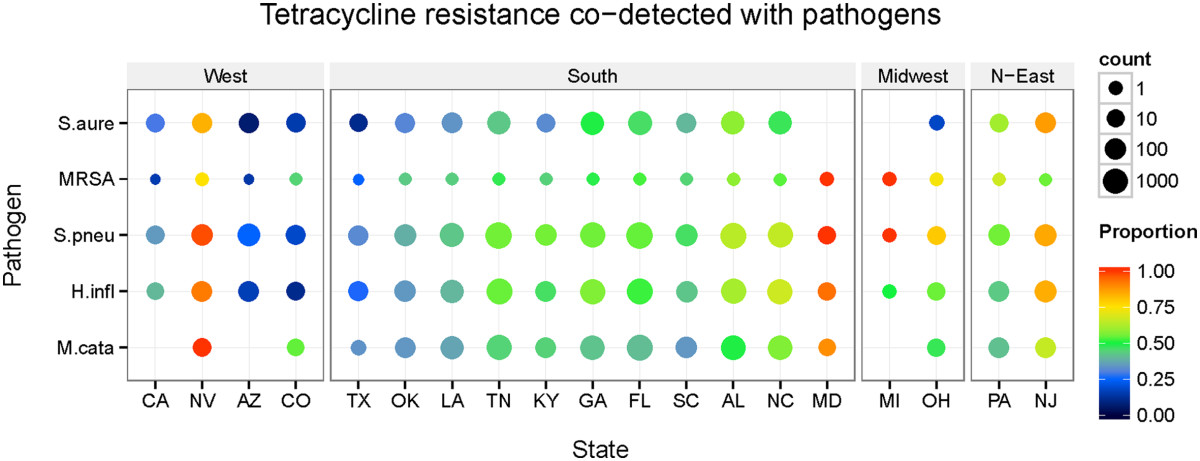
**Heatmap Displaying the Longitudinal Effect of TRG Co-detection across the United States.** The heat map is ordered by state on the bottom where the eastern states are on the right and the western states are on the left side of the map. The circles which are most red are the states that have the highest levels of tetracycline resistance co-detection regarding each pathogen, while states that are the most blue have the lowest rates. Size of the circles correspond to sample size of the data.

Figure 3 includes a heatmap indicating the co-detection frequencies of TRGs and each of the 5 respiratory pathogens separated by state from November 2009- August 2013. Additional file 1: Table S9 displays the number of positive tests recorded from each state and the frequency that the pathogen was co-detected with a TRG. The highest frequency of co-detection occurred in the Northeast, especially Maryland and New Jersey. In the Southeast, Florida, Alabama, and Georgia accounted for the largest percentage of TRG co-detection. Along a westward gradient, the percentages of TRG co-detection dropped dramatically. Louisiana, Oklahoma, Texas, and Colorado all had co-detection frequencies under 53%. The level of TRGs co-detected from patients in New Jersey for the five pathogens was between 54.4% and 86.9%. Moving westward, the frequencies of TRGs co-detected was lower in Tennessee with levels between 42.7% and 55.3% for the five pathogens. In Texas, the rate of co-detection decreased even further to between 33.3% and 10.7%. Since Eastern states reported a much higher frequency of co-detection over Western states, a longitudinal trend was established.

## Discussion

Figure 1 indicates that the frequency of co-detection between the 5 respiratory pathogens and TRGs continues to rise over time. One trend observed in Figure 1 is the periodicity, where the co-detection rates of TRGs and each pathogen reach a maximum during the middle of each year. The frequency of co-detection is the highest in the summer and the lowest in the winter of each year. Various literature report a seasonal incidence of antibiotic resistance in various pathogens [31–33]. It is possible that this trend occurs from fluctuation in the number of tests physicians administer to patients during different times of the year. Alternatively, the seasonal trend here may be occurring because of the different levels of antibiotics prescribed to patients throughout the each season, especially during the flu season [33]. The CDC considers the respiratory illness/flu season in the United States to begin in October and end in May, peaking in February [34]. As a result of the rise in number of antibiotics prescribed during February, an increased number of pathogens activate the production of TRGs [35]. Throughout spring months, TRG up-regulation and transmission continues to increase until months after flu season subsides. As a result, the rise in TRG co-detection frequency seen each summer could be a delayed effect from the increase in tetracycline exposure from the rise in number of antibiotic prescriptions that are administered during early winter/spring of each year. Moreover, these seasonal data could also have a clinical impact as drug therapy could be periodically altered and antibiotic effectiveness could be increased. Physicians may have greater success prescribing tetracycline in the winter when co-detection frequency is lower, than in the summer when co-detection is at maximum levels. Seasonal trends could be occurring with other first-line antibiotics as well, but more studies are needed to test this hypothesis.

Figure 2 illustrates that all 4 age groups rose in co-detection frequency over time, but the frequencies of co-detection follow different trends for the age groups. Across the 5 pathogens, patients age (0–2] display the lowest frequency of co-detection of TRGs and respiratory pathogens, while patients age (13–50] demonstrate the highest rate of co-detection. These data suggest that tetracycline usage can be optimized in different age groups over the yearly cycle. Even though tetracycline has the highest chance of being effective in patients age (0–2], physicians typically avoid prescribing tetracycline to younger children, as the treatment can cause staining of permanent teeth [36, 37]. Once dental maturity is reached, the staining of teeth from tetracycline use is no longer an issue. The avoidance of prescribing tetracycline to infant patients could be an underlying reason for the lower co-detection measurement in the youngest age bracket. Alternatively, one approved use of tetracycline is to combat dermatological pathogens and is a therapeutic option for treating young adults with acne [31, 34]. The increased utilization of tetracycline in teenage patients could be an underlying cause for the higher TRG co-detection frequency observed in the (13–50] age group. In addition, environmental factors such as diet may have an effect on the large co-detection frequency of both (13–50] and (50–100] age groups. Since over half the antibiotics produced for use in the United States are utilized for agricultural purposes, it may be plausible to hypothesize that the longer a person ingests tetracycline-infused foods, the greater likelihood that normal microbial flora build an immunity to the antibiotic. Then, when a respiratory pathogen infects a patient, these flora can pass tetracycline genes to the foreign pathogen via conjugation [38].There are two possible explanations for why a longitudinal effect is observed in Figure 3. Physicians in the Northeast could be prescribing tetracycline to patients at higher rates than in other areas of the country. Alternatively, various diets and varieties of food eaten across the United States could have an impact on different frequencies of TRG and respiratory pathogen co-detection. An underlying reason for the varying frequencies of TRG co-detection throughout different regions of the United States could be because of geographical differences regarding tetracycline application in agriculture. The only outlier in the Western states is Nevada, which has one of the largest rates of co-detection, but is surrounded by states that have the lowest levels of co-detection of TRGs and respiratory pathogens. There is no apparent hypothesis for why Nevada has a large co-detection frequency. Taken together, these data suggest an environmental influence, but a subsequent analysis of data estimating per-state tetracycline loads would be needed to correlate these data to co-detection frequencies.

There are two common causes for increased tetracycline exposure. The first includes excess prescriptions being provided to patients by physicians and the other involves overexposure in agriculture and livestock feed. It is likely that these pathogens contained genes and pathways that predated the antibiotic era that could neutralize tetracycline [39, 40]. Increased utilization in the fields of agriculture and medicine allowed these time-weighted genetic determinants such as synthesis of inactivation enzymes, efflux pumps, or ribosomal protection proteins to be up-regulated and passed to other pathogens via plasmid transfer [39–42]. *S. aureus*, MRSA, *S. pneumonia, H. influenzae,* and *M. catarrhalis* are all found in proximity in the upper respiratory tract of most humans, therefore, it is plausible that these pathogens exchange genetic information frequently. The genomes of many pathogens contain TRGs with the three major genes including tet (K), tet (M), and tet (O) [1, 15, 16]. In this study, only genetic targets for tet (K) and tet (M) were included in testing. With the exclusion of tet (O) and other tet genes, it can be speculated that the actual rate of tetracycline resistance in patients is even higher than seen here.

A strong hypothesis for the high frequencies of TRG co-detection is that the high tetracycline use in agriculture facilitates tetracycline resistant gene activations, which is then passed to other pathogens via conjugation. The rampant use of antibiotics is a more pressing issue in the United States than in other countries because over half the antibiotics that are produced in the United States are used for agricultural purposes, an extremely high percentage [3, 4]. For many years it has been disputed whether or not antibiotic use in agriculture is responsible for the rise in the number of antibiotic resistant pathogens. The FDA argues antibiotic levels recommended for agricultural use have been tested in a laboratory setting on bacteria in culture, with very low frequencies of pathogens acquiring resistance [43]. However, some literature demonstrates that sub-inhibitory antibiotic exposure can cause development of resistance over time [44–47]. Recently, the FDA has begun to regulate the amount of antibiotics utilized in the agricultural and medical industries [9]. Playing close attention to the levels of pathogens that are becoming resistant to antibiotics is imperative because throughout the past 15 years only 9 new antibiotics have been approved to combat certain illnesses [8]. Since there are a limited number of new antibiotic medications in the developmental pipeline, it is important to monitor levels of tetracycline resistant pathogens so that the medication can continue to be an effective treatment for acne and uncomplicated community-acquired MRSA [48–50].

## Conclusion

After a data mining analysis, it is clear that there is a relationship between *S. aureus*, MRSA, *S. pneumoniae, H. influenzae,* and *M.catarrhalis* and the co-detection of TRGs. Since 2010, there has been a rise in TRG co-detection by at least 24% for each of the five potential respiratory pathogens studied and co-detection frequency is more widespread in summer months than in winter months. Patients aged (13–50] show the highest rate of TRG co-detection with respiratory pathogens, while patients aged (0–2] show the lowest frequency. These findings could correlate with the deleterious effect of tetracycline prescriptions given to younger children because of medication side-effects or the large amount of teenagers who are prescribed tetracycline for the effective treatment of acne. Partitioned by geography, the Northeastern United States demonstrated the largest percentage of TRG co-detection, while co-detection rates decreased longitudinally westward.

The CDC has realized that antibiotic resistance is the largest threat to future global health and these data further underscore the need for careful management of antibiotics. By establishing certain trends regarding tetracycline resistance, we can begin to uncover underlying causes for the rise in TRG co-detection frequency. These data are consistent with the hypothesis that environmental pressures in medicine and agriculture effect levels of TRGs. As a result, many people who have been educated on the overuse of antibiotics in society have switched to strict organic diets so that the risk of coming in contact with TRGs is minimized. Moreover, the FDA, CDC, and Department of Agriculture are beginning to revise regulations regarding antibiotic usage. The overuse of antibiotics in society is an ongoing controversial topic. However, the continuous monitoring of co-detection rates between pathogens and antibiotic resistance genes is imperative so that physicians can assess the effectiveness of an antibiotic at the time of infection and provide optimal patient care.

Further studies are needed to generalize these descriptive findings to a larger population and to associate environmental factors with the rise in tetracycline resistance. We hope that this study fosters a rich discussion of this vitally important medical topic.

### Ethics statement

This study was approved by Chesapeake Internal Review Board (IRB) (Columbia, MD) which has full accreditation with the Association for the Accreditation of Human Research Protection Programs (AAHRPP) (Protocol #: Pro00010357). Using the Department of Health and Human Services regulations at 45 CFR 46, the IRB determined that this study does not constitute human subject research and, therefore, does not require IRB oversight. All procedures employed followed the guidelines in the Declaration of Helsinki and HIPAA. This study analyzed a dataset containing patient age, gender, U.S. state, test date, and test results. Patient identity was not available to the researchers in this study.

## Electronic supplementary material

Additional file 1: Table S1: Percentages of Positive Tests Detected with TRGs aggregated by Year. The table includes sample size, number of tests, number of positive tests co-detected with TRGs, and % of positive tests co-detected with TRGs for each year and pathogen. **Table S2.** Percentage of Tests Positive for *S. aureus* and TRGs Categorized by Age and Year. **Table S3.** Percentage of Tests Positive for MRSA and TRGs Categorized by Age and Year. **Table S4.** Percentage of Tests Positive for *S. pneumoniae* and TRGs Categorized by Age and Year. **Table S5.** Percentage of Tests Positive for *H. influenzae* and TRGs Categorized by Age and Year. **Table S6.** Percentage of Tests Positive for *M. catarrhalis* and TRGs Categorized by Age and Year. Each table (Tables S2-S6) includes the sample size, number of positive tests, number of tests co-detected with a TRG, and % of tests co-detected for a TRG for each pathogen in this study that is categorized by age group and year. **Table S7.** Percentage of Positive Tests detected with TRGs Categorized by State. The table includes the sample size, number of positive tests, number of tests co-detected with a TRG, and % of tests co-detected for a TRG for each pathogen in this study organized by state. (PDF 509 KB)

Below are the links to the authors’ original submitted files for images.

Authors’ original file for figure 1

Authors’ original file for figure 2

Authors’ original file for figure 3

Authors’ original file for figure 4

Authors’ original file for figure 5

Authors’ original file for figure 6

## Abbreviations

CDC: Center for disease control and prevention
FDA: Food and drug administration
RES: Respiratory panel
TEM-PCR: Target enriched multiplex polymerase chain reaction
TRGs: Tetracycline resistance genes.
