# Supplementary material for: The frequency of tetracycline resistance genes co-detected with respiratory pathogens: a database mining study uncovering descriptive trends throughout the United States
Source: BMC Infect Dis. 2014 Aug 25;14:460. doi: 10.1186/1471-2334-14-460 (PMC4156627; doi:10.1186/1471-2334-14-460)
Supplement: Supplementary file 1 — Additional file 1: Table S1: Percentages of Positive Tests Detected with TRGs aggregated by Year. The table includes sample size, number of tests, number of positive tests co-detected with TRGs, and % of positive tests co-detected with TRGs for each year and pathogen. Table S2. Percentage of Tests Positive for S. aureus and TRGs Categorized by Age and Year. Table S3. Percentage of Tests Positive for MRSA and TRGs Categorized by Age and Year. Table S4. Percentage of Tests Positive for S. pneumoniae and TRGs Categorized by Age and Year. Table S5. Percentage of Tests Positive for H. influenzae and TRGs Categorized by Age and Year. Table S6. Percentage of Tests Positive for M. catarrhalis and TRGs Categorized by Age and Year. Each table (Tables S2-S6) includes the sample size, number of positive tests, number of tests co-detected with a TRG, and % of tests co-detected for a TRG for each pathogen in this study that is categorized by age group and year. Table S7. Percentage of Positive Tests detected with TRGs Categorized by State. The table includes the sample size, number of positive tests, number of tests co-detected with a TRG, and % of tests co-detected for a TRG for each pathogen in this study organized by state. (PDF 509 KB) [file 12879_2014_3763_MOESM1_ESM.pdf]

Table S1: Percentages of Positive Tests Detected with TRGs aggregated by Year

		S. aureus	MRSA	*S. pneumoniae*	*H. influenzae*	*M. catarrhalis*
2009 (N= 6,396)	Number of + Tests	578	106	458	173	N/A
	Number of + Tests co-detected with TRGs	23	7	94	25	N/A
	% of + Tests co-detected with TRGs	**3.98%**	**6.60%**	**20.52%**	**14.45%**	**N/A**
2010 (N= 11,256)	Number of + Tests	1,079	604	1,677	1,236	N/A
	Number of + Tests co-detected with TRGs	271	203	641	348	N/A
	% of + Tests co-detected with TRGs	**25.12%**	**33.61%**	**38.22%**	**28.16%**	**N/A**
2011 (N= 14,914)	Number of + Tests	1,385	1,361	3,554	2,383	1,630
	Number of + Tests co-detected with TRGs	571	518	1,575	1,015	427
	% of + Tests co-detected with TRGs	**41.23%**	**38.06%**	**44.32%**	**42.59%**	**26.20%**
2012 (N= 26,841)	Number of + Tests	1,276	2,315	6,793	5,158	5,517
	Number of + Tests co-detected with TRGs	706	1,269	3,886	2,925	2,378
	% of + Tests co-detected with TRGs	**55.33%**	**54.82%**	**57.21%**	**56.71%**	**43.10%**
2013 (N= 20,834)	Number of + Tests	667	1,789	5,631	5,331	5,344
	Number of + Tests co-detected with TRGs	454	1,069	3,654	3,230	2,699
	% of + Tests co-detected with TRGs	**68.07%**	**59.75%**	**64.89%**	**60.59%**	**50.51%**

Table S2: Percentage of Tests Positive for *S. aureus* and TRGs Categorized by Age and Year

		2009	2010	2011	2012	2013
S. aureus tested patients aged (0-2]	Total Number of Patient Tests (N)	516	1,405	3,725	7,601	6,207
	Number of + Tests	17	96	323	272	129
	Number of + Tests co-detected with TRGs	2	32	114	127	77
	% of + Tests co-detected with TRGs	**11.76%**	**33.33%**	**35.29%**	**46.69%**	**59.69%**
S. aureus tested patients aged (2-13]	Total Number of Patient Tests (N)	1,187	2,273	4,451	10,198	7,501
	Number of + Tests	139	250	552	696	342
	Number of + Tests co-detected with TRGs	5	54	214	385	244
	% of + Tests co-detected with TRGs	**3.60%**	**21.60%**	**38.77%**	**55.32%**	**71.35%**
S. aureus tested patients aged (13-50]	Total Number of Patient Tests (N)	3,532	5,103	3,849	5,546	3,986
	Number of + Tests	357	542	346	245	142
	Number of + Tests co-detected with TRGs	13	131	185	169	107
	% of + Tests co-detected with TRGs	**3.64%**	**24.17%**	**53.47%**	**68.98%**	**75.35%**
S. aureus tested patients aged (50-100]	Total Number of Patient Tests (N)	1,161	2,475	2,889	3,496	3,140
	Number of + Tests	65	191	164	63	49
	Number of + Tests co-detected with TRGs	3	54	58	32	26
	% of + Tests co-detected with TRGs	**4.62%**	**28.27%**	**35.37%**	**50.79%**	**53.06%**

Table S3: Percentage of Tests Positive for MRSA and TRGs Categorized by Age and Year

		2009	2010	2011	2012	2013
MRSA tested patients aged (0-2]	Total Number of Patient Tests (N)	516	1,405	3,725	7,601	6,207
	Number of + Tests	7	52	204	591	541
	Number of + Tests co-detected with TRGs	0	24	108	346	330
	% of + Tests co-detected with TRGs	**0.00%**	**46.15%**	**52.94%**	**58.54%**	**61.00%**
MRSA tested patients aged (2-13]	Total Number of Patient Tests (N)	1,187	2,273	4,451	10,198	7,501
	Number of + Tests	13	112	459	1,082	796
	Number of + Tests co-detected with TRGs	1	29	154	539	443
	% of + Tests co-detected with TRGs	**7.69%**	**25.89%**	**33.55%**	**49.82%**	**55.65%**
MRSA tested patients aged (13-50]	Total Number of Patient Tests (N)	3,532	5,103	3,849	5,546	3,986
	Number of + Tests	53	262	347	423	269
	Number of + Tests co-detected with TRGs	3	94	155	268	181
	% of + Tests co-detected with TRGs	**5.66%**	**35.88%**	**44.67%**	**63.36%**	**67.29%**
MRSA tested patients aged (50-100]	Total Number of Patient Tests (N)	1,161	2,475	2,889	3,496	3,140
	Number of + Tests	33	178	271	219	183
	Number of + Tests co-detected with TRGs	3	56	101	116	115
	% of + Tests co-detected with TRGs	**9.09%**	**31.46%**	**37.27%**	**52.97%**	**62.84%**

Table S4: Percentage of Tests Positive for *S. pneumoniae* and TRGs Categorized by Age and Year

		2009	2010	2011	2012	2013
S. pneumoniae tested patients aged (0-2]	Total Number of Patient Tests (N)	516	1,405	3,725	7,601	6,207
	Number of + Tests	156	575	1,473	2,847	2,417
	Number of + Tests co-detected with TRGs	40	214	561	1,362	1,339
	% of + Tests co-detected with TRGs	**25.64%**	**37.22%**	**38.09%**	**47.84%**	**55.40%**
S. pneumoniae tested patients aged (2-13]	Total Number of Patient Tests (N)	1,187	2,273	4,451	10,198	7,501
	Number of + Tests	225	640	1,446	3,139	2,455
	Number of + Tests co-detected with TRGs	35	201	571	1,856	1,660
	% of + Tests co-detected with TRGs	**15.56%**	**31.41%**	**39.49%**	**59.13%**	**67.62%**
S. pneumoniae tested patients aged (13-50]	Total Number of Patient Tests (N)	3,532	5,103	3,849	5,546	3,986
	Number of + Tests	59	308	404	557	503
	Number of + Tests co-detected with TRGs	15	153	294	475	446
	% of + Tests co-detected with TRGs	**25.42%**	**49.68%**	**72.77%**	**85.28%**	**88.67%**
S. pneumoniae tested patients aged (50-100]	Total Number of Patient Tests (N)	1,161	2,475	2,889	3,496	3,140
	Number of + Tests	18	154	231	250	256
	Number of + Tests co-detected with TRGs	4	73	149	193	209
	% of + Tests co-detected with TRGs	**22.22%**	**47.40%**	**64.50%**	**77.20%**	**81.64%**

Table S5: Percentage of Tests Positive for *H. influenzae* and TRGs Categorized by Age and Year

		2009	2010	2011	2012	2013
H. influenzae tested patients aged (0-2]	Total Number of Patient Tests (N)	516	1,405	3,725	7,601	6,207
	Number of + Tests	46	283	888	1,824	1,977
	Number of + Tests co-detected with TRGs	10	92	285	788	985
	% of + Tests co-detected with TRGs	**21.74%**	**32.51%**	**32.09%**	**43.20%**	**49.82%**
H. influenzae tested patients aged (2-13]	Total Number of Patient Tests (N)	1,187	2,273	4,451	10,198	7,501
	Number of + Tests	73	457	1,072	2,633	2,593
	Number of + Tests co-detected with TRGs	9	109	450	1,604	1,663
	% of + Tests co-detected with TRGs	**12.33%**	**23.85%**	**41.98%**	**60.92%**	**64.13%**
H. influenzae tested patients aged (13-50]	Total Number of Patient Tests (N)	3,532	5,103	3,849	5,546	3,986
	Number of + Tests	37	373	286	509	522
	Number of + Tests co-detected with TRGs	4	106	199	407	429
	% of + Tests co-detected with TRGs	**10.81%**	**28.42%**	**69.58%**	**79.96%**	**82.18%**
H. influenzae tested patients aged (50-100]	Total Number of Patient Tests (N)	1,161	2,475	2,889	3,496	3,140
	Number of + Tests	17	123	137	192	239
	Number of + Tests co-detected with TRGs	2	41	81	126	153
	% of + Tests co-detected with TRGs	**11.76%**	**33.33%**	**59.12%**	**65.63%**	**64.02%**

Table S6: Percentage of Tests Positive for *M. catarrhalis* and TRGs Categorized by Age and Year

		2011	2012	2013
M. catarrhalis tested patients aged (0-2]	Total Number of Patient Tests (N)	3,725	7,601	6,207
	Number of + Tests	815	2,955	2,857
	Number of + Tests co-detected with TRGs	196	1,208	1,357
	% of + Tests co-detected with TRGs	**24.05%**	**40.88%**	**47.50%**
M. catarrhalis tested patients aged (2-13]	Total Number of Patient Tests (N)	4,451	10,198	7,501
	Number of + Tests	650	2,214	2,119
	Number of + Tests co-detected with TRGs	169	971	1,091
	% of + Tests co-detected with TRGs	**26.00%**	**43.86%**	**51.49%**
M. catarrhalis tested patients aged (13-50]	Total Number of Patient Tests (N)	3,849	5,546	3,986
	Number of + Tests	103	213	206
	Number of + Tests co-detected with TRGs	39	129	144
	% of + Tests co-detected with TRGs	**37.86%**	**60.56%**	**69.90%**
M. catarrhalis tested patients aged (50-100]	Total Number of Patient Tests (N)	2,889	3,496	3,140
	Number of + Tests	62	135	162
	Number of + Tests co-detected with TRGs	25	70	107
	% of + Tests co-detected with TRGs	**40.32%**	**51.85%**	**66.05%**

Table S7: Percentage of Positive Tests detected with TRGs Categorized by State

		S. aureus	MRSA	*S. pneumoniae*	*H. influenzae*	*M. catarrhalis*
AL (N=10,124)	Number of + Tests	544	942	2,395	2,027	1,601
	Number of + Tests co-detected with TRGs	315	547	1,527	1,237	795
	% of + Tests co-detected with TRGs	**57.90%**	**58.07%**	**63.76%**	**61.03%**	**49.66%**
AZ (N=6,258)	Number of + Tests	633	173	714	445	N/A
	Number of + Tests co-detected with TRGs	43	25	179	72	N/A
	% of + Tests co-detected with TRGs	**6.79%**	**14.45%**	**25.07%**	**16.18%**	**N/A**
CA (N=403)	Number of + Tests	52	13	49	20	N/A
	Number of + Tests co-detected with TRGs	15	2	17	8	N/A
	% of + Tests co-detected with TRGs	**28.85%**	**15.38%**	**34.69%**	**40.00%**	**N/A**
CO (N=1,738)	Number of + Tests	144	49	193	176	13
	Number of + Tests co-detected with TRGs	22	22	36	19	7
	% of + Tests co-detected with TRGs	**15.28%**	**44.90%**	**18.65%**	**10.80%**	**53.85%**
FL (N=15,752)	Number of + Tests	1,084	1,465	4,439	3,585	4,106
	Number of + Tests co-detected with TRGs	505	759	2,393	1,837	1,677
	% of + Tests co-detected with TRGs	**46.59%**	**51.81%**	**53.91%**	**51.24%**	**40.84%**
GA (N=7,876)	Number of + Tests	499	613	1,955	1,637	1,344
	Number of + Tests co-detected with TRGs	248	301	1,075	928	563
	% of + Tests co-detected with TRGs	**49.70%**	**49.10%**	**54.99%**	**56.69%**	**41.89%**
KY (N=974)	Number of + Tests	47	68	165	141	115
	Number of + Tests co-detected with TRGs	15	30	91	66	51
	% of + Tests co-detected with TRGs	**31.91%**	**44.12%**	**55.15%**	**46.81%**	**44.35%**
LA (N= 3,826)	Number of + Tests	176	378	1,074	772	736
	Number of + Tests co-detected with TRGs	58	165	456	305	268
	% of + Tests co-detected with TRGs	**32.95%**	**43.65%**	**42.46%**	**39.51%**	**36.41%**
MD (N= 60)	Number of + Tests	0	1	11	15	9
	Number of + Tests co-detected with TRGs	0	1	11	14	8
	% of + Tests co-detected with TRGs	**0.00%**	**100.00%**	**100.00%**	**93.33%**	**88.89%**
NC (N= 8,529)	Number of + Tests	589	427	1750	1384	1023
	Number of + Tests co-detected with TRGs	283	226	1138	921	579
	% of + Tests co-detected with TRGs	**48.05%**	**52.93%**	**65.03%**	**66.55%**	**56.60%**
NJ (N=782)	Number of + Tests	61	44	185	163	101
	Number of + Tests co-detected with TRGs	53	24	157	137	66
	% of + Tests co-detected with TRGs	**86.89%**	**54.55%**	**84.86%**	**84.05%**	**65.35%**
NV (N=721)	Number of + Tests	54	36	86	61	15
	Number of + Tests co-detected with TRGs	45	27	84	56	15
	% of + Tests co-detected with TRGs	**83.33%**	**75.00%**	**97.67%**	**91.80%**	**100.00%**
OH (N= 261)	Number of + Tests	11	15	24	22	29
	Number of + Tests co-detected with TRGs	2	11	19	12	14
	% of + Tests co-detected with TRGs	**18.18%**	**73.33%**	**79.17%**	**54.55%**	**48.28%**
OK (N= 1,805)	Number of + Tests	112	156	425	300	181
	Number of + Tests co-detected with TRGs	35	67	163	103	62
	% of + Tests co-detected with TRGs	**31.25%**	**42.95%**	**38.35%**	**34.33%**	**34.25%**
PA (N= 573)	Number of + Tests	33	48	156	137	162
	Number of + Tests co-detected with TRGs	20	32	86	59	67
	% of + Tests co-detected with TRGs	**60.61%**	**66.67%**	**55.13%**	**43.07%**	**41.36%**
SC (N=1,749)	Number of + Tests	88	163	352	258	293
	Number of + Tests co-detected with TRGs	35	73	165	109	100
	% of + Tests co-detected with TRGs	**39.77%**	**44.79%**	**46.88%**	**42.25%**	**34.13%**
TN (N= 16,958)	Number of + Tests	705	1,518	3,911	2,944	2,730
	Number of + Tests co-detected with TRGs	301	736	2,162	1,601	1,219
	% of + Tests co-detected with TRGs	**42.70%**	**48.48%**	**55.28%**	**54.38%**	**44.65%**
TX (N= 1,124)	Number of + Tests	103	36	132	137	6
	Number of + Tests co-detected with TRGs	11	9	42	35	2
	% of + Tests co-detected with TRGs	**10.68%**	**25.00%**	**31.82%**	**25.55%**	**33.33%**
VA (N= 426)	Number of + Tests	27	7	38	15	N/A
	Number of + Tests co-detected with TRGs	4	0	14	0	N/A
	% of + Tests co-detected with TRGs	**14.81%**	**0.00%**	**36.84%**	**0.00%**	**N/A**
