# Supplementary figures and images for: The frequency of tetracycline resistance genes co-detected with respiratory pathogens: a database mining study uncovering descriptive trends throughout the United States
Source: BMC Infect Dis. 2014 Aug 25;14:460. doi: 10.1186/1471-2334-14-460 (PMC4156627; doi:10.1186/1471-2334-14-460)

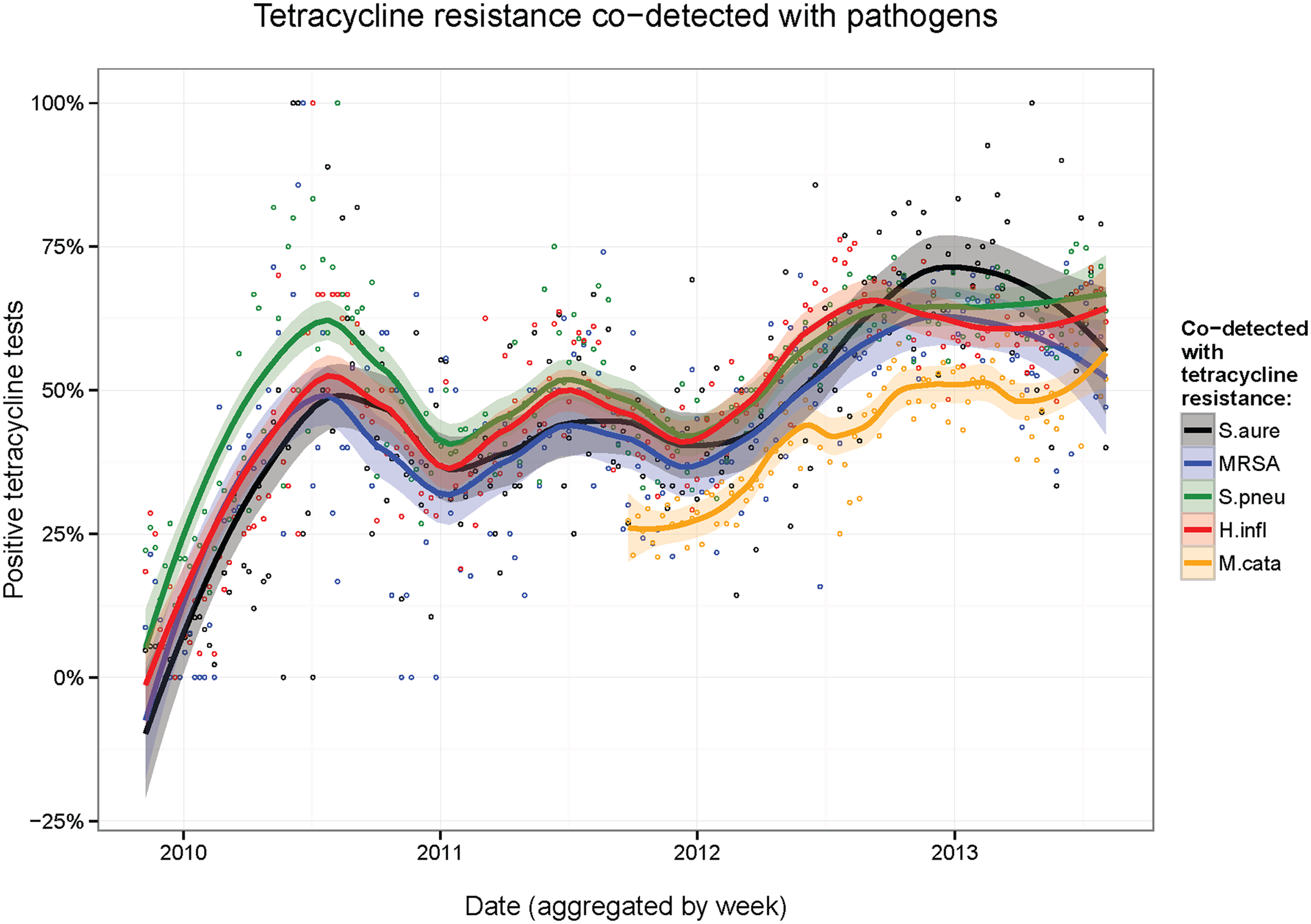

Supplement: Supplementary file 2 — Authors’ original file for figure 1 [file 12879_2014_3763_MOESM2_ESM.tiff]

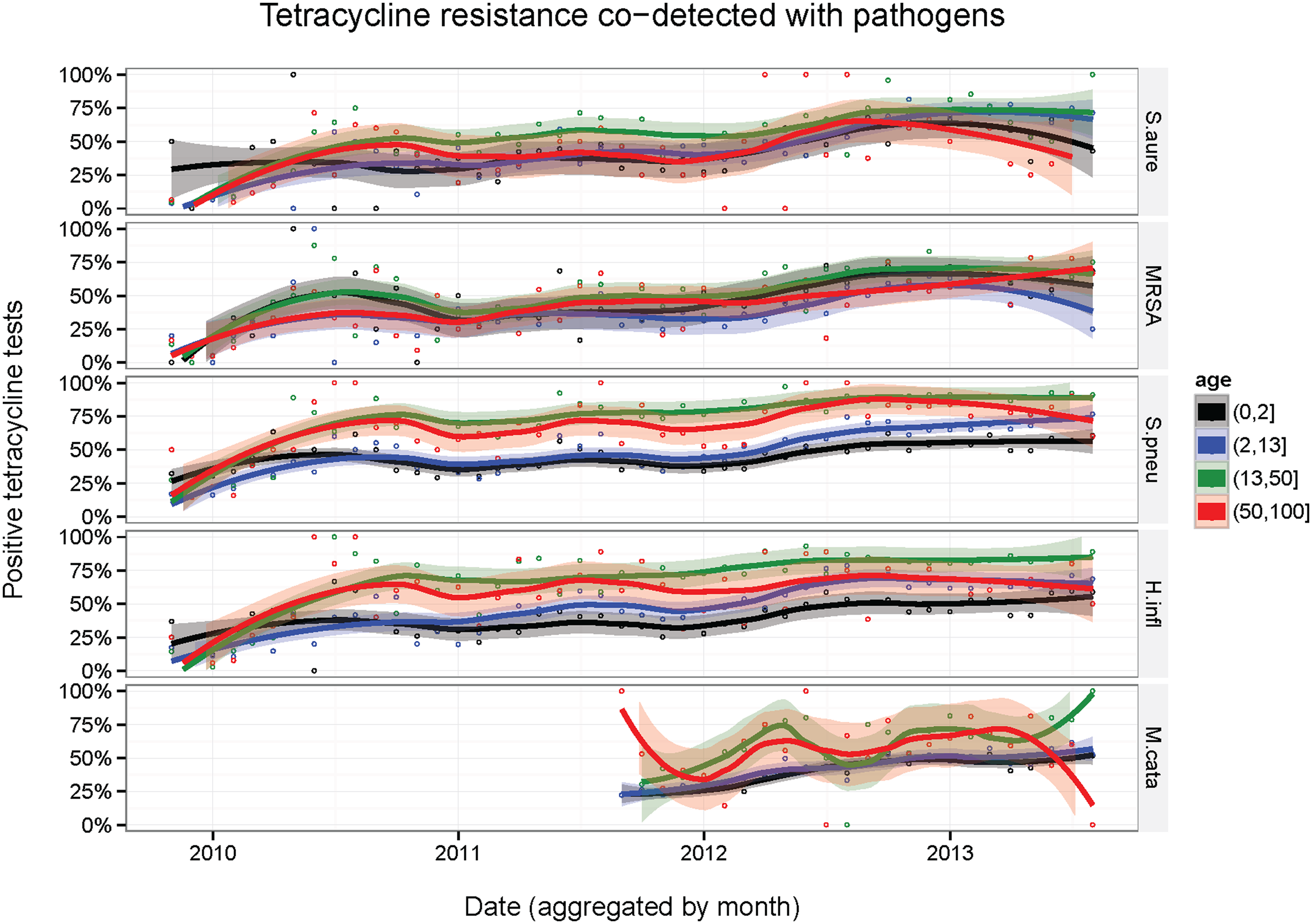

Supplement: Supplementary file 3 — Authors’ original file for figure 2 [file 12879_2014_3763_MOESM3_ESM.tiff]

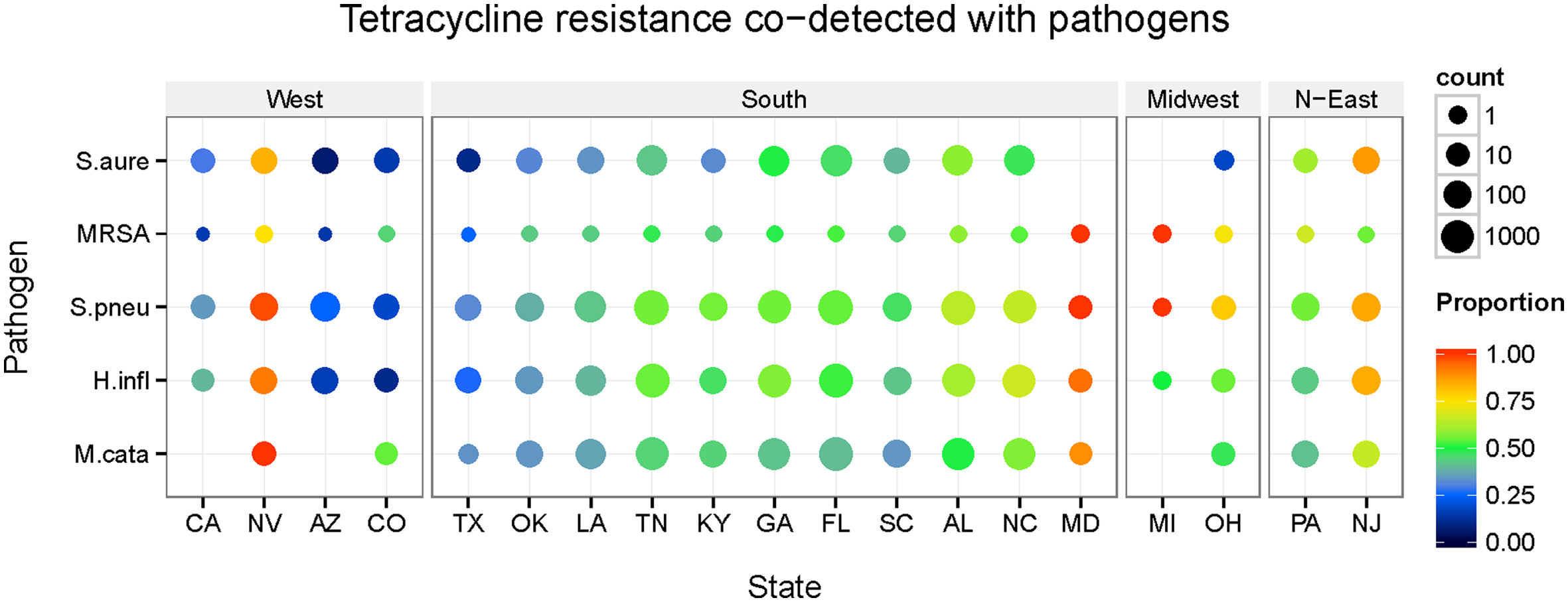

Supplement: Supplementary file 4 — Authors’ original file for figure 3 [file 12879_2014_3763_MOESM4_ESM.tiff]
