# Supplementary material for: The frequency of tetracycline resistance genes co-detected with respiratory pathogens: a database mining study uncovering descriptive trends throughout the United States
Source: BMC Infect Dis. 2014 Aug 25;14:460. doi: 10.1186/1471-2334-14-460 (PMC4156627; doi:10.1186/1471-2334-14-460)
Supplement: Supplementary file 5 — Authors’ original file for figure 4 [file 12879_2014_3763_MOESM5_ESM.pdf]

Age Groups- % positive for ***S. aureus*** co-detected with TRGs	2010	2011	2012	2013
0-2 years old	33.73	35.57	49.39	64.62
2-13 years old	21.69	38.73	55.1	75.67
13-50 years old	24.12	53.62	65.98	84.29
50< years old	0	34.97	50.79	59.1

Age Groups- % positive for MRSA co-detected with TRGs	2010	2011	2012	2013
0-2 years old	0	38.34	59.41	70.41
2-13 years old	25.93	33.55	49.72	64.57
13-50 years old	0	36	58.1	69.13
50< years old	31.46	37.69	53.64	68.39

Age Groups- % positive for ***S. pneumoniae*** co-detected with TRGs	2010	2011	2012	2013
0-2 years old	37.23	38.36	47.6	55.1
2-13 years old	31.5	39.36	59.5	68.1
13-50 years old	49.67	72.41	85.11	89.3
50< years old	48	66.8	77.1	82.8

Age Groups- % positive for ***H. influenzae*** co-detected with TRGs	2010	2011	2012	2013
0-2 years old	33	32.6	42.5	51
2-13 years old	23.87	42	61	66.4
13-50 years old	28.42	68.8	85.6	82.11
50< years old	33.6	59	64.44	63.77

Age Groups- % positive for ***M. catarrhalis*** co-detected with TRGs	2010	2011	2012	2013
0-2 years old	0	24.5	40.76	47.93
2-13 years old	0	26	43.71	52.33
13-50 years old	36.02	45.66	64.12	73.67
50< years old	0	40.3	50.4	64.8
