# Supplementary material for: The frequency of tetracycline resistance genes co-detected with respiratory pathogens: a database mining study uncovering descriptive trends throughout the United States
Source: BMC Infect Dis. 2014 Aug 25;14:460. doi: 10.1186/1471-2334-14-460 (PMC4156627; doi:10.1186/1471-2334-14-460)
Supplement: Supplementary file 6 — Authors’ original file for figure 5 [file 12879_2014_3763_MOESM6_ESM.pdf]

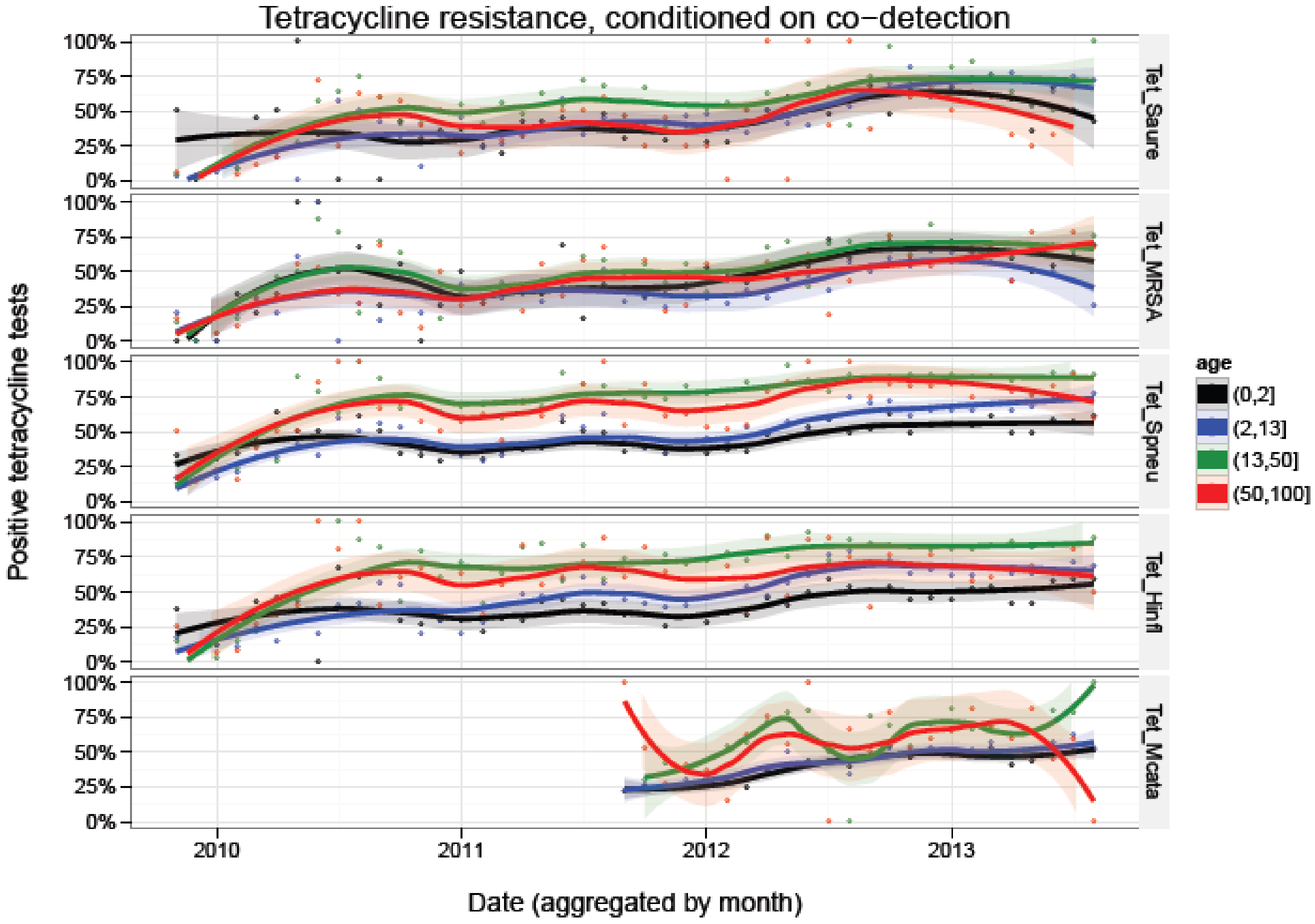

Figure 2: The tables on the top correspond to the scatterplot on the bottom. The tables have percentages of each patient co-detected with TRGs and a pathogen; broken down by year into 4 specific age groups. Each age group has an increasing antibiotic resistance co-detection rate from 2010-2013. The graphs on the bottom have a curved best fit line that cuts through data points aggregated by month. 95% confidence intervals are also recorded in the glowing color around each line. The trend for each pathogen is that infants ages 0-2 have the smallest rate of tetracycline resistance co-detection, while patients from the age 13-50 have the highest percentage. *S. aureus* and MRSA have similar co-detection between some age groups, but the age group of 13-50 still appears to have the highest rate of co-detection and age groups 0-2 and 2-13 with the lowest rates.
