# Supplementary material for: The frequency of tetracycline resistance genes co-detected with respiratory pathogens: a database mining study uncovering descriptive trends throughout the United States
Source: BMC Infect Dis. 2014 Aug 25;14:460. doi: 10.1186/1471-2334-14-460 (PMC4156627; doi:10.1186/1471-2334-14-460)
Supplement: Supplementary file 7 — Authors’ original file for figure 6 [file 12879_2014_3763_MOESM7_ESM.pdf]

% of Pathogens Co-detected with TRGs 10'-13'	CA	NV	CO	TX	OK	LA	SC	FL	GA	MS	AL	TN	KY	IN	MD	OH	PA	NJ
S. aureus	22.34	63.64	12.94	8.8	37.59	32.72	48.67	56.09	55.15	38.46	63.76	48.26	32.3	75	88.46	44.83	67.09	87.2
MRSA	11.11	63.83	48.37	22.22	47.34	50.89	51.11	59.27	54.98	59.38	64.12	56.01	50	53.33	85.71	68.29	73.83	69.61
S. pneumoniae	34.69	80	18.97	24.71	38.67	40.88	46.67	54.68	54.43	34.48	64.04	55.67	52.8	72.72	91.3	63.16	55.17	84.1
H. influenzae	40	79.16	10.73	22.72	34.6	38.83	43.61	52.32	56.68	22.22	62.47	54.66	48.15	73.68	95.45	46.67	47.01	85.2
M. catarrhalis	0	100	50	33.33	33.67	36.41	35.2	41.78	42.37	25	50.82	45.05	42.86	60.71	84.61	43.75	42.28	66.38

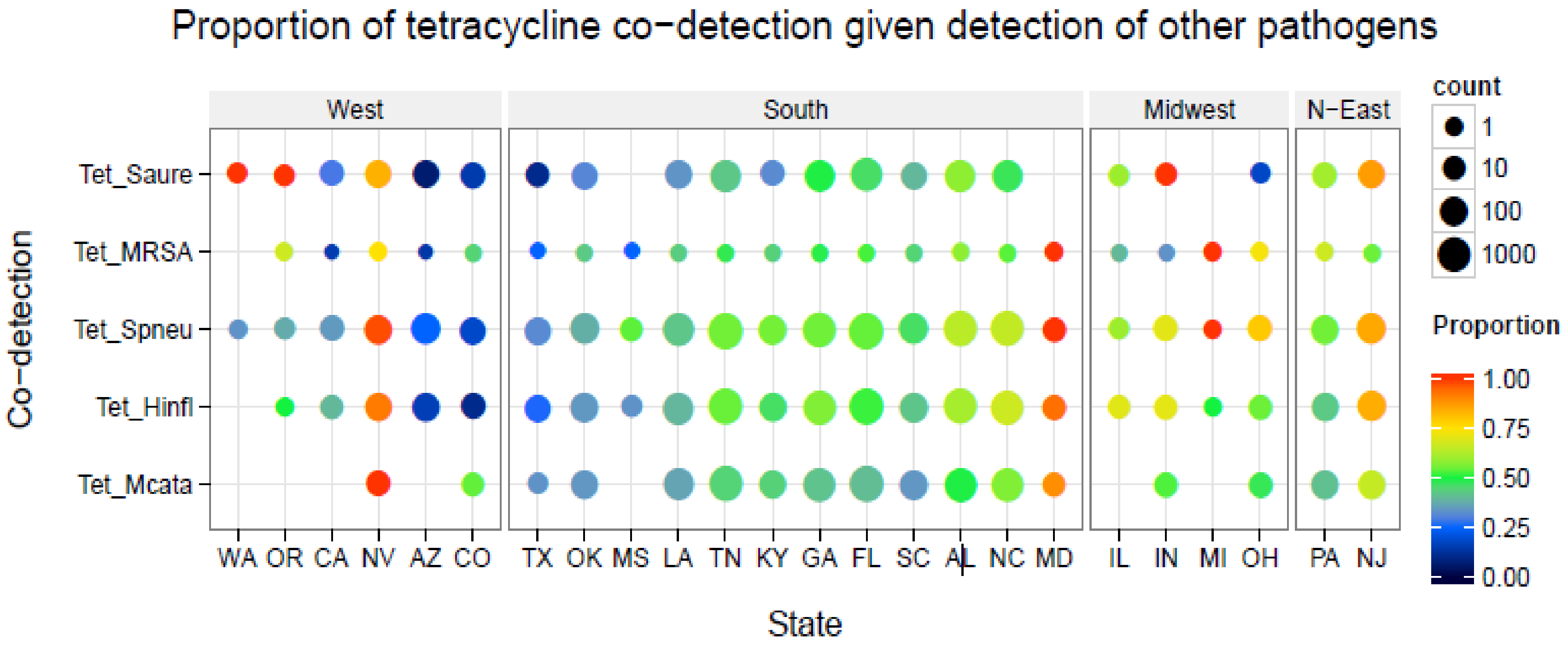

Figure 3: The information in the table above matches the data in the heat map below. The heat map is ordered by state on the bottom where the eastern region of states is on the right and the west is on the left. The circles which are most red are the states that have the highest levels of tetracycline resistance co-detection with each bacteria, while states that are the most blue have the lowest rates. There seems to be a longitudinal effect in data meaning that eastern most states are the most red, while the colors fade to blue in the west. Size of the circles correspond to sample size of the data. Some states were omitted from the table so that it would fit on the page.
